# Genome-wide placental DNA methylation analysis of severely growth-discordant monochorionic twins reveals novel epigenetic targets for intrauterine growth restriction

**DOI:** 10.1186/s13148-016-0238-x

**Published:** 2016-06-21

**Authors:** Maian Roifman, Sanaa Choufani, Andrei L. Turinsky, Sascha Drewlo, Sarah Keating, Michael Brudno, John Kingdom, Rosanna Weksberg

**Affiliations:** Division of Clinical and Metabolic Genetics, The Hospital for Sick Children, Toronto, Ontario Canada; Department of Paediatrics, University of Toronto, Toronto, Ontario Canada; Genetics and Genome Biology Program, The Hospital for Sick Children, Toronto, Ontario Canada; The Prenatal and Medical Genetics Program, Department of Obstetrics and Gynaecology, Mount Sinai Hospital, Toronto, Ontario Canada; Centre for Computational Medicine, The Hospital for Sick Children, Toronto, Ontario Canada; C.S. Mott Center for Human Growth and Development, Wayne State School of Medicine, Wayne State University, Detroit, MI USA; Maternal-Fetal Medicine Division, Department of Obstetrics and Gynecology, Mount Sinai Hospital, Toronto, Ontario Canada; Department of Laboratory Medicine and Pathology, University of Toronto, Toronto, Ontario Canada; Department of Computer Science, University of Toronto, Toronto, Ontario Canada; Department of Obstetrics and Gynecology, University of Toronto, Toronto, Ontario Canada; Institute of Medical Science, University of Toronto, Toronto, Ontario Canada

**Keywords:** Intrauterine growth restriction, Monozygotic twins, Monochorionic twins, DNA methylation, Wnt pathway, Cadherin pathway, *DECR1*, *ZNF300*, *LEPR*

## Abstract

**Background:**

Intrauterine growth restriction (IUGR), which refers to reduced fetal growth in the context of placental insufficiency, is etiologically heterogeneous. IUGR is associated not only with perinatal morbidity and mortality but also with adult-onset disorders, such as cardiovascular disease and diabetes, posing a major health burden. Placental epigenetic dysregulation has been proposed as one mechanism that causes IUGR; however, the spectrum of epigenetic pathophysiological mechanisms leading to IUGR remains to be elucidated. Monozygotic monochorionic twins are particularly affected by IUGR, in the setting of severe discordant growth. Because monozygotic twins have the same genotype at conception and a shared maternal environment, they provide an ideal model system for studying epigenetic dysregulation of the placenta.

**Results:**

We compared genome-wide placental DNA methylation patterns of severely growth-discordant twins to identify novel candidate genes for IUGR. Snap-frozen placental samples for eight severely growth-discordant monozygotic monochorionic twin pairs were obtained at delivery from each twin. A high-resolution DNA methylation array platform was used to identify methylation differences between IUGR and normal twins. Our analysis revealed differentially methylated regions in the promoters of eight genes: *DECR1*, *ZNF300*, *DNAJA4*, *CCL28*, *LEPR*, *HSPA1A/L*, *GSTO1*, and *GNE*. The largest methylation differences between the two groups were in the promoters of *DECR1* and *ZNF300*. The significance of these group differences was independently validated by bisulfite pyrosequencing, implicating aberrations in fatty acid beta oxidation and transcriptional regulation, respectively. Further analysis of the array data identified methylation changes most prominently affecting the Wnt and cadherin pathways in the IUGR cohort.

**Conclusions:**

Our results suggest that IUGR in monozygotic twins is associated with impairments in lipid metabolism and transcriptional regulation as well as cadherin and Wnt signaling. We show that monozygotic monochorionic twins discordant for growth provide a useful model to study one type of the epigenetic placental dysregulation that drives IUGR.

**Electronic supplementary material:**

The online version of this article (doi:10.1186/s13148-016-0238-x) contains supplementary material, which is available to authorized users.

## Background

Fetal growth restriction is considered a disorder with significant etiologic heterogeneity, as there are many known fetal and maternal causes of growth restriction [1]. When growth restriction occurs in the context of placental insufficiency, it is termed intrauterine growth restriction (IUGR). IUGR, which affects 10–15 % of pregnancies [2], is associated not only with significant perinatal morbidity and mortality [2] but also with adult-onset diseases, such as cardiovascular disease and diabetes [3–5]. Thus, IUGR poses a major human health burden. The association between IUGR and adult-onset disease is thought to result from fetal programming, wherein fetal adaptive mechanisms “set” metabolic regulatory pathways in response to fetal environments, such as limited nutrition. Consequently, in a society where postnatal nutrient supply exceeds the prenatally “sensed” demands, these metabolic set points predispose the organism to disease later in life [3–5]. The molecular basis of fetal programming is likely to be mediated by epigenetic mechanisms such as DNA methylation.

Early embryonic development depends on normally functioning epigenetic modifications such as DNA methylation, to guide spatial and temporal gene activation and repression that mediate normal growth, cell differentiation and morphogenesis [6]. Likewise, normal placental growth requires appropriate epigenetic regulation of gene expression.

The spectrum of epigenetic pathophysiological mechanisms leading to IUGR remains to be elucidated. These epigenetic alterations, which can involve the fetus and/or the placenta, may affect expression of imprinted or non-imprinted genes important for normal fetal growth and development. Imprinted genes are normally expressed from a single allele in a parent of origin-specific manner [6]. This expression pattern is coordinated via CpG-rich differentially methylated regions (DMRs) in imprinted domains. Loss of imprinting, characterized by aberrant methylation of an imprinted DMR, may restrict the growth of both the placenta and the fetus, causing IUGR, as in the undergrowth disorder Russell-Silver syndrome (OMIM 180860) [6].

IUGR due to placental insufficiency typically exhibits specific diagnostic vascular and pathological placental features. It has been proposed that the intrauterine environment can affect placental development and alter placental gene expression, thereby reducing the placenta’s ability to support optimal fetal growth. Studies investigating placental epigenetic dysregulation have identified altered DNA methylation of genes involved in placental development, including trophoblast differentiation, angiogenesis, and endocrine signaling [7]. Placental epigenetic dysregulation, in particular altered DNA methylation, has been proposed as a mechanism underlying the aberrant gene expression in the pathophysiology of IUGR [7].

Monochorionic (MC) twins are monozygotic (MZ; i.e., identical) twins that share the same placenta. They provide an ideal model for studying epigenetic regulation of the placenta because of their identical genotypes and shared maternal environment. Monochorionic twins are also particularly affected by IUGR in the setting of severe discordant growth, which occurs in 20 % of MC pregnancies [8, 9]. Severe discordant growth is a major contributor to perinatal morbidity and mortality in MC twin pregnancies. Growth discordance is 2.5-fold higher in MC versus dichorionic twin pregnancies [10]. IUGR in the setting of severe growth discordance of MC twins is typically characterized by unequal placental share, where the smaller twin has a smaller share of the placenta [11]. A better understanding of the placental dysfunction underlying MC growth discordance not only holds promise for improving postnatal outcomes in this population but will also offer clues to understanding IUGR on a wider scale in singletons and the molecular basis of fetal programming relevant to increased risks for adult-onset disease.

In this study, we compared genome-wide placental DNA methylation (DNAm) patterns of severely growth-discordant MC twins. Our microarray analysis identified novel candidate differentially methylated regions for IUGR, including those overlapping the promoters of three genes *DECR1*, *LEPR*, and *ZNF300*. These differences were statistically significant when validated by an independent methodology, bisulfite pyrosequencing. A pathway analysis supported a role for epigenetic dysregulation in IUGR pathogenesis of the Wnt and cadherin pathways, which are known to be important in embryonic development. While a single prior study reported targeted gene expression in growth-discordant MC twins [12], the current study is the first genome-wide investigation of DNAm in MC growth-discordant twins.

## Results

We sought to identify novel candidate genes for IUGR by comparing genome-wide placental DNAm using Illumina HumanMethylation450 arrays of eight severely growth-discordant MC twin pairs. The clinical characteristics of the MZ twins in this study are detailed in Additional file 1: Table S1. Growth-discordant twin pairs had birth weight differences between 21 and 59 %. All growth-restricted twins measured below the tenth centile in weight for gestational age, while their normal weight counterparts measured above the tenth centile.

A description of placental pathology for these twins is found in Additional file 1: Table S2. Pathological features typically associated with IUGR, such as velamentous cord insertion, advanced villous maturity, thrombosis, and infarction, were consistently present in the IUGR-affected twin’s placental share.

### Genome-wide DNA methylation patterns

**Fig. 1:**
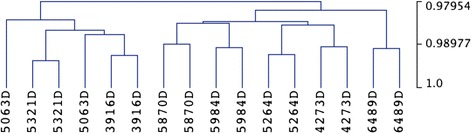
Genome-wide unsupervised hierarchical clustering. Correlation between global DNA methylation patterns involving 399,118 autosomal CpGs was highest between co-twins for 7/8 discordant twin pairs, with the twin pair 5063D being the only exception. This suggests that IUGR is not associated with genome-wide DNA methylation changes. Monozygosity was molecularly confirmed for all twin pairs. Pearson correlation with average linkage was used as the clustering metric, with the scale shown on the right. Samples 4273D, 5264D, 5063D, and 5321D overlap with samples from a previous study [12]

Differential DNAm analysis was performed on a total of 399,118 sites after quality control analysis of the data (see the “Methods” section). When comparing global DNAm patterns using unsupervised hierarchical clustering, twins were most similar to their own co-twins in 7/8 discordant twin pairs (Fig. 1). This suggests that IUGR is not associated with genome-wide DNAm changes, a finding previously described in other epigenome-wide association studies [13, 14].

### Differentially methylated regions associated with IUGR

**Fig. 2:**
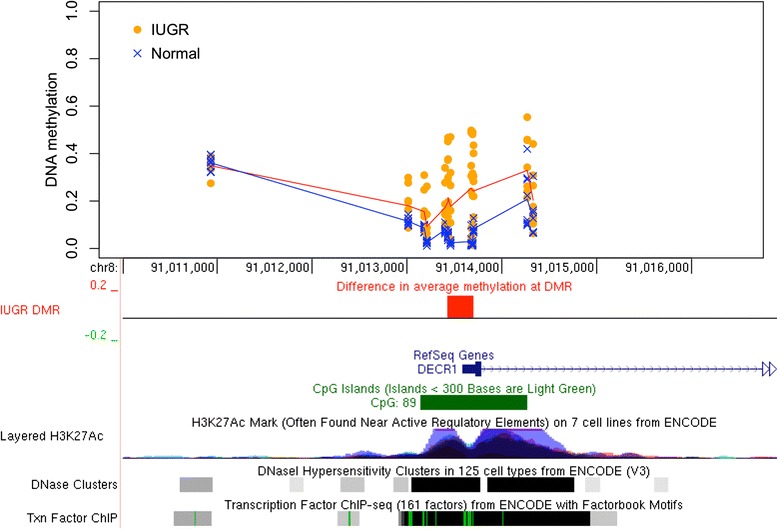
DNAm changes in IUGR twins in *DECR1* promoter. Methylation levels in IUGR twins (*orange circles*) and unaffected co-twins (*blue crosses*) are shown, arranged vertically for each CpG in the region spanning the *DECR1* gene promoter. The mean methylation levels are shown for the IUGR cohort (*red line*) and controls (*blue line*). The top six CpGs with methylation change above 10 %, and no more than 500 bp apart, comprise the DMR (*red box*), which also corresponds to the wider gap between the *red* and *blue lines*. Other genomic features, such as CpG islands, histone marks, DNase hypersensitivity clusters, and transcription factor binding sites are also shown. Visualization is provided by the UCSC Genome Browser and was inspired by DMRcate software [66]

**Fig. 3:**
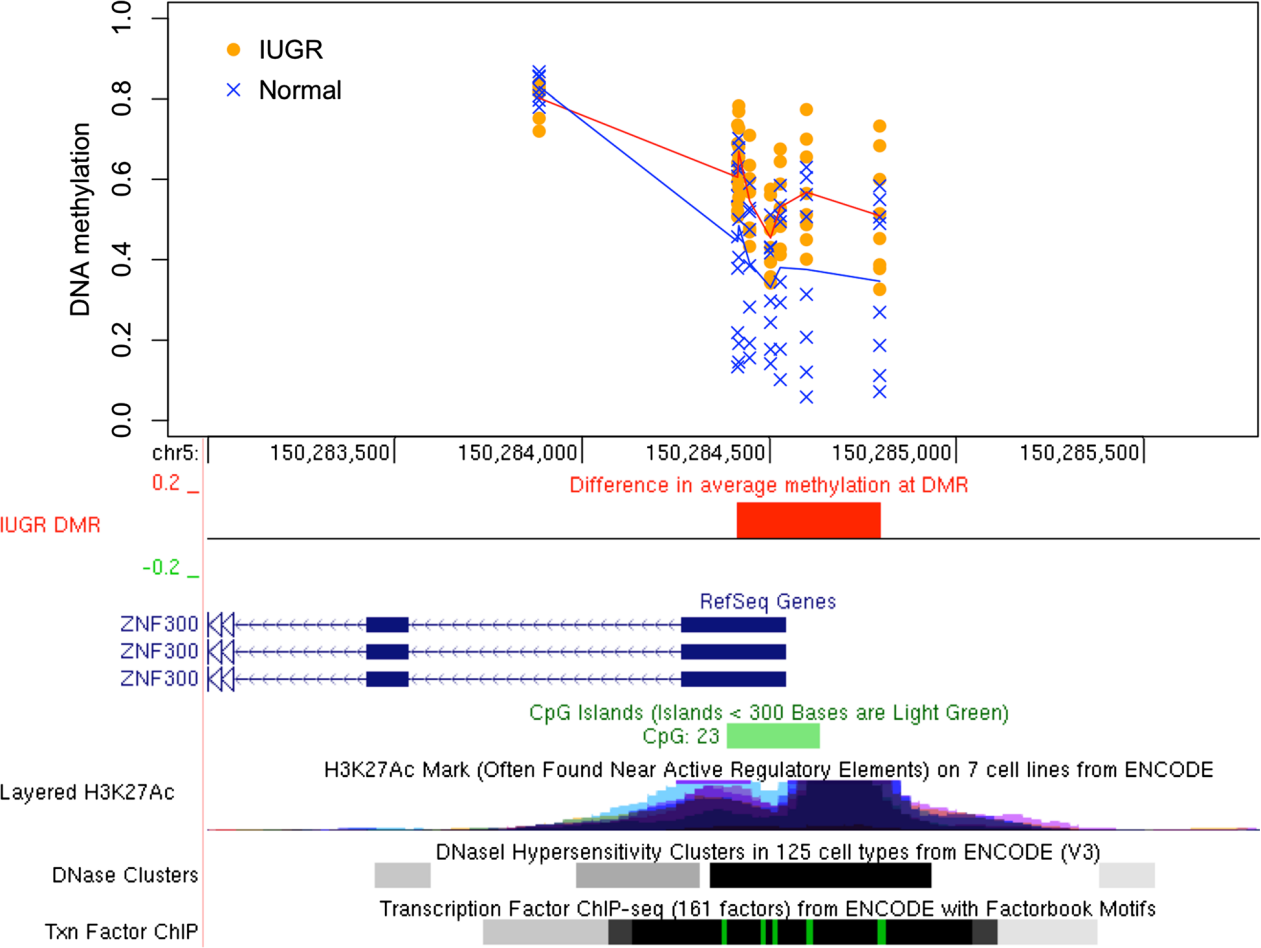
DNAm changes in IUGR twins in *ZNF300* promoter. Methylation levels in IUGR twins (*orange circles*) and unaffected co-twins (*blue crosses*) are shown, arranged vertically for each CpG in the region spanning the promoter of the *ZNF300* gene transcript. The mean methylation levels are shown for the IUGR cohort (*red line*) and controls (*blue line*). The top seven CpGs with methylation change above 10 % comprise the DMR (*red box*), which also corresponds to the wider gap between the *red* and *blue lines*

**Fig. 4:**
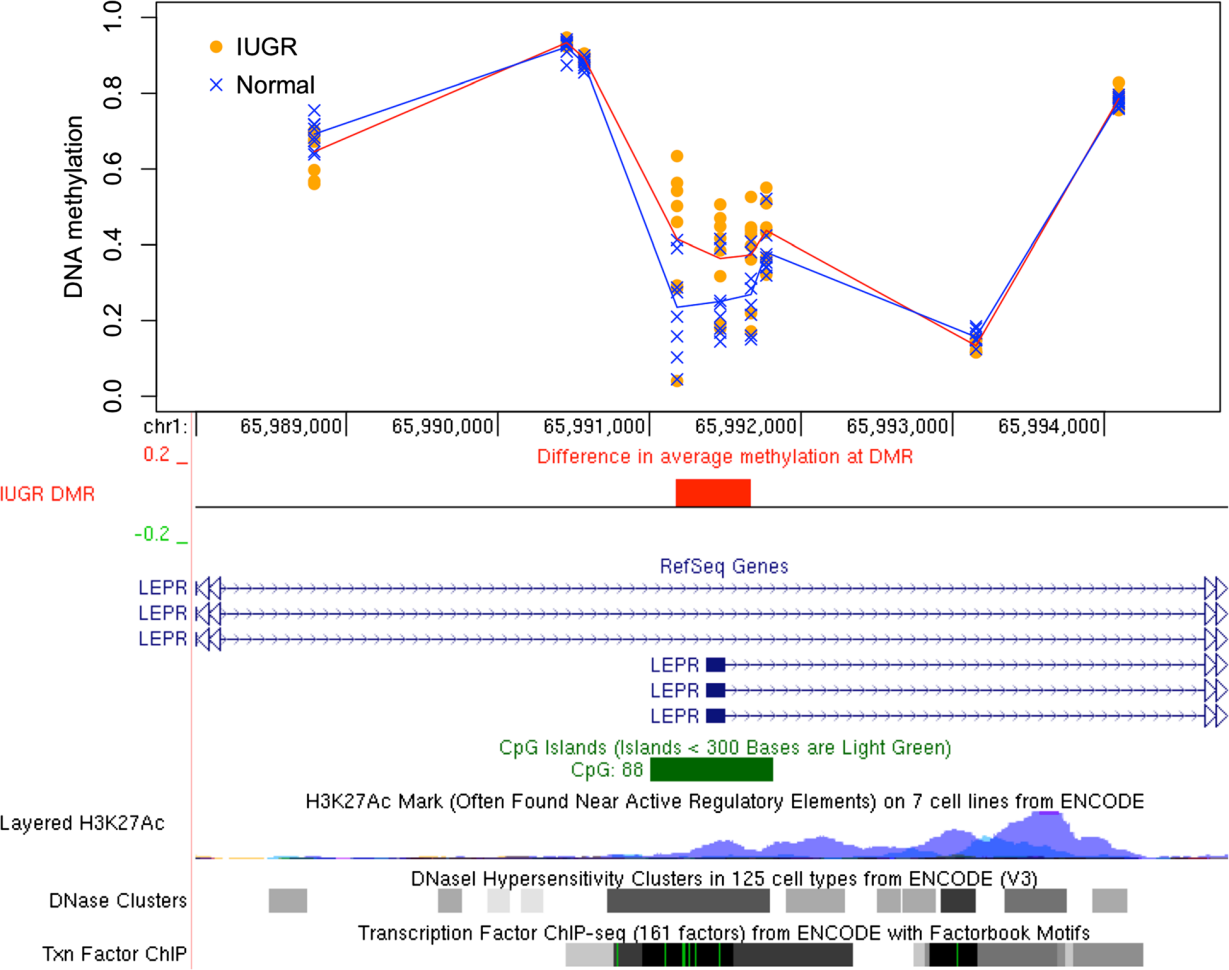
DNAm changes in IUGR twins in *LEPR* promoter. Methylation levels in IUGR twins (*orange circles*) and unaffected co-twins (*blue crosses*) are shown, arranged vertically for each CpG in the region spanning the promoter of one of the *LEPR* gene transcripts. The mean methylation levels are shown for the IUGR cohort (*red line*) and controls (*blue line*). The top three CpGs with methylation change above 10 % comprise the DMR (*red box*), which also corresponds to the wider gap between the *red* and *blue lines*

To find genomic regions with DNAm differences in IUGR, we used bump hunting [15], which is known to improve the statistical power and robustness by combining methylation patterns across nearby CpG sites in smaller datasets [16]. After accounting for the potential confounding effects of the twins’ sex, gestational age, and maternal age (see the “Methods” section), our analysis revealed eight statistically significant (*p* < 0.05) DMRs where the magnitude of the DNAm differences between IUGR and the other twin exceeded 10 % (Table 1). These DMRs match the promoters of eight genes (listed in decreasing order of methylation difference between IUGR and controls): *DECR1*, *ZNF300*, *DNAJA4*, *CCL28*, *LEPR*, *HSPA1A/L*, *GSTO1*, *GNE* (Table 1). For six of eight genes, the IUGR twin demonstrated a gain of DNAm. Inspection of individual DMRs revealed consistent patterns of DNAm change that also corresponded well with other epigenetic features, such as histone marks, DNase hypersensitivity regions, and transcription factor binding sites (Figs. 2, 3, 4; Additional file 1: Figures S1–S2).

**Table 1:** Differentially methylated regions (DMR). Shown are the DMRs with the significance level *p* < 0.05 as reported by the *bumphunter* software and spanning at least three CpGs with the average DNA methylation differences (delta beta) between IUGR and healthy twins above 10 % by magnitude in all CpGs. The DMRs are sorted by the magnitude of delta-beta differences. DMR length is measured in basepairs. Gene matches represent the overlaps between the DMR and the gene’s body or promoter region up to 1500 bp from the transcription start site. Genome coordinates are given with respect to hg19 genome assembly

Chr	Start	End	Δβ	*p* value	CpGs	Length	Gene
8	91013426	91013697	0.175	0.0027	6	272	DECR1
5	150284416	150284796	0.161	0.0032	7	381	ZNF300
15	78556834	78556945	−0.152	0.0082	5	112	DNAJA4
5	43396953	43397288	−0.139	0.0081	6	336	CCL28
1	65991176	65991664	0.133	0.0329	3	489	LEPR
6	31783322	31783482	0.130	0.0069	7	161	HSPA1A/L
10	106014615	106014893	0.126	0.0401	3	279	GSTO1
9	36258114	36258885	0.120	0.0114	6	772	GNE

### *DECR1*, *ZNF300*, and *LEPR* DNA methylation results

Three of the eight genes identified in the bump hunting analysis were chosen for validation by targeted bisulfite pyrosequencing (PyroMark Q24; Qiagen) (see the “Methods” section). *DECR1* and *ZNF300* were selected because they exhibited the largest methylation differences in our analysis, and *LEPR* was chosen because of its recent association with IUGR in a separate MZ-MC twin study [12].

The largest methylation differences were found in the promoter region of the *DECR1* gene, which encodes 2,4 dienoyl-CoA reductase (DECR), a mitochondrial enzyme in the unsaturated fatty acid beta oxidation pathway vital for unsaturated fat metabolism.

Placental samples from the IUGR twins exhibited a gain of methylation in all of the CpG sites within the CpG island located in the *DECR1* promoter region, compared to their normal weight counterparts (Fig. 2) (Table 1). The mean DNAm differences in IUGR (also known as delta beta or Δβ) across all twin pairs for the *DECR1* was 17.5 % (bump hunting *p* = 0.0027), reaching as high as 22.5 and 21.4 %, respectively, for the top two differentially methylated CpG sites within the DMR. There was a very substantial gain in DNAm levels in five of eight discordant twin pairs, ranging on average between 14 and 42 % across the DMR, and a small gain in one other pair (4 %). The remaining two twin pairs showed no difference in DNAm (less than 1 % change); see Additional file 1: Figure S3 for details.

**Fig. 5:**
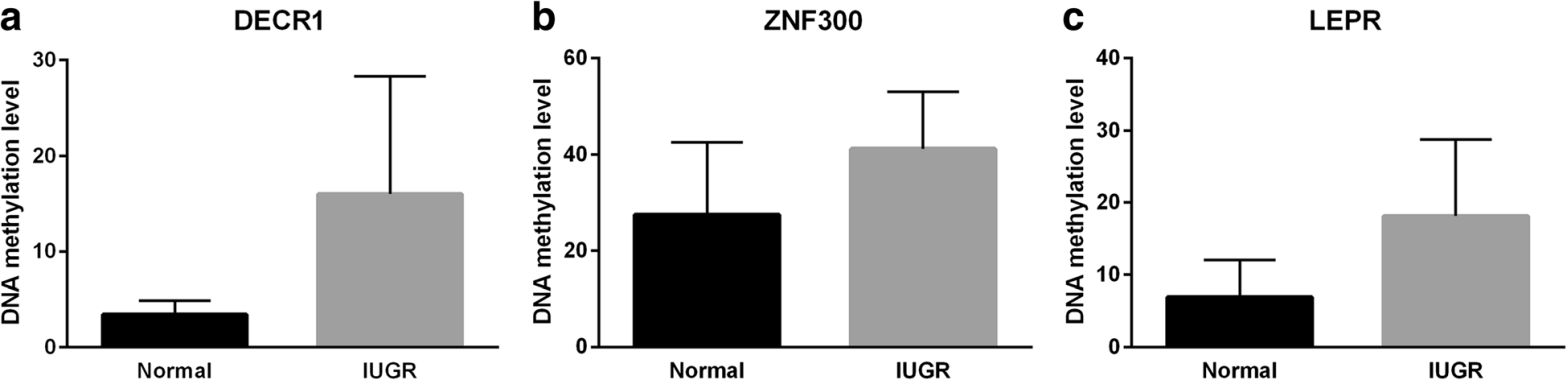
Validation of candidate IUGR regions by bisulfite pyrosequencing. Bar charts representing the DNA methylation levels at two CpG sites overlapping *DECR1* (cg01971612, cg18485485) (**a**), three CpG sites overlapping *ZNF300* (cg04675542, cg02343823, cg08580836) (**b**), and one CpG site overlapping *LEPR* (cg08234308) (**c**) in both IUGR (*gray color*) and healthy co-twins (*black color*)

The results obtained for the two top *DECR1*-promoter CpGs, cg01971612 and cg18485485, were independently validated through bisulfite pyrosequencing analysis in the same set of twins (Fig. 5a). A significant difference in DNAm (mean Δβ gain of 12.6 %) was observed in the IUGR compared to the normal twin group for both CpG sites (paired *t* test, two-tailed, *p* = 0.021) (Additional file 1: Figure S4, see the “Methods” section).

Following *DECR1*, the next largest DMR showing 16 % gain of methylation in IUGR (*p* = 0.0032) was located in the promoter of the zinc finger protein-coding gene, *ZNF300*, (Fig. 3), which plays an important role in tumor growth and immune modulation. Five of the twin pairs showed a methylation gain between 18 and 32 % in IUGR at this DMR, with two more pairs showing small or negligible gains (5.4 and 1.3 %, respectively). The remaining one pair showed a small DNAm loss of 4.3 %; see Additional file 1: Figure S5 for details.

The results for three CpG sites (cg04675542, cg02343823, cg08580836) within the *ZNF300* DMR were independently validated using targeted bisulfite pyrosequencing analysis (Fig. 5b). Significant differences were demonstrated for the three CpG sites in IUGR compared to the normal twin group (mean Δβ gain of 13.7 %; paired *t* test, two-tailed, *p* = 0.016) (Additional file 1: Figure S6).

In the *LEPR* gene, placental samples from the IUGR twins exhibited a gain of methylation at all CpG sites located near the promoter region (Fig. 4). The DMR showed an average DNAm gain of 13.3 % (*p* = 0.033) in IUGR twins compared to their healthy counterparts, with four of eight discordant twin pairs having very substantial gain of between 13 and 33 %. A small gain was found in three other pairs (between 3.4 and 6.4 %); the remaining one pair showed negligible DNAm difference (1.7 % change); see Additional file 1: Figure S7 for details.

DNAm levels for the top CpG site (cg08234308) within the *LEPR* DMR were independently validated using targeted bisulfite pyrosequencing analysis (Fig. 5c) and demonstrated a significant difference (mean Δβ gain of 11.2 %; paired *t* test, two-tailed, *p* = 0.022) between the IUGR and the normal twin groups (Additional file 1: Figure S8).

### Differentially methylated CpG sites associated with IUGR

**Fig. 6:**
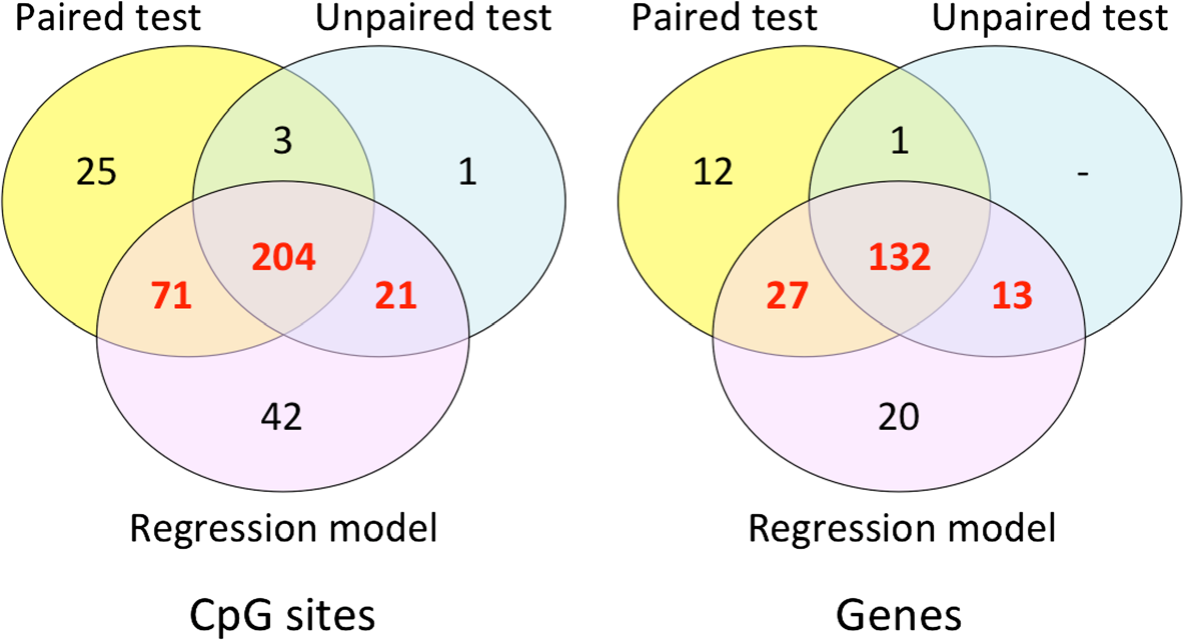
CpG sites used for functional enrichment analysis. Only the CpGs that have a DNAm difference at least 10 % in IUGR twins were considered. A set of 296 CpGs (numbers in *red*) was selected as the sites that were identified by *limma* regression analysis after accounting for confounding effects and also passed at least one of the non-parametric tests for IUGR association. The *right* Venn diagram shows the number of genes matching each criteria of significance, where 172 genes comprise the set tested for functional enrichment

**Fig. 7:**
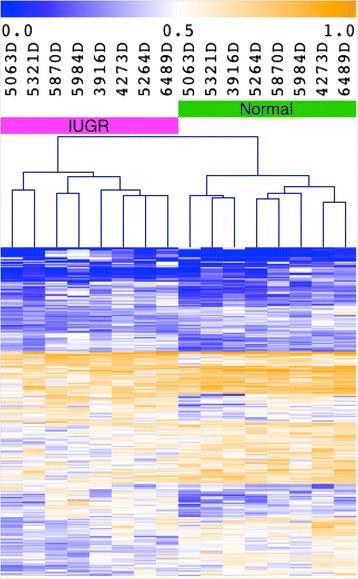
The putatively affected CpGs are able to separate the IUGR cohort from the normal cohort. Clustering heatmap shows IUGR (*magenta*) and normal (*green*) twin samples, shown in columns, forming two separate clusters in Manhattan distance metric. Rows represent the 296 affected CpGs. The *color scale* at the *top* indicates the range of the DNAm values

To assess the broader functional implications of the genome-wide DNAm changes in IUGR, our analysis was extended to the level of individual CpGs (see the “Methods” section). This analysis identified 296 statistically significant CpG sites (Fig. 6), which overlap 172 genes or gene-promoter regions (Additional file 2: Table S3). As an additional validation step, using these 296 CpGs to quantify the methylation profiles resulted in the IUGR cohort clustering separately from the group of eight unaffected twins (Fig. 7). This suggested that the identified collection of CpGs which overlap the list of eight genomic regions identified by bump hunting is also sufficiently representative of methylation differences in IUGR, and is therefore also likely to reflect broad functional patterns related to the disorder.

### Functional enrichment

Enrichment analysis showed a significant over-representation of functional groups highly relevant to the IUGR phenotype, such as fetal development, intracellular signaling, and cell adhesion. For example, 55 of the 172 genes were involved in “anatomical structure development” (*p* = 1.0e−7 after false discovery rate (FDR) correction) and 40 genes were associated with “nervous system development” (corrected *p* = 2.6e−10). As many as 70 genes, or 41 % of our list, were annotated as “intrinsic to membrane” (corrected *p* = 0.011). Other enriched functional groups were related to cell adhesion, developmental processes, and regulation of transcription (Additional file 2: Table S4). We also analyzed the enrichment of the 296 individual CpG sites in the context of much wider genomic regions, extending up to 100,000 bp to the nearest gene, which facilitated the functional assignment of intergenic CpGs. The functional categories detected in this analysis were related to developmental processes, lipid metabolism (e.g., lipopolysaccharide metabolic process, response to cholesterol) and cellular response to various stimuli (Additional file 2: Table S5).

The enriched functional groups such as “intrinsic to membrane,” “anatomical structure development” and “nervous system development” also included most of the genes from the Wnt and cadherin pathways. Analysis of pathway enrichment confirmed that the cadherin pathway, which mediates calcium-ion-dependent cell adhesion, was prominent among the candidate genes (20 genes, *p* = 5.9e−10 after FDR correction), as was the related Wnt signaling pathway (23 affected genes, *p* = 5.0e−8 after FDR correction).

## Discussion

To our knowledge, this is the first report of genome-wide DNA methylation analysis in growth-discordant MC twins. None of the top eight genes identified in our analysis is associated with a known imprinted region, implicating a mechanism of IUGR that is distinct from those impacted in imprinting disorders. Two of the eight genes differentially methylated in the IUGR group (*DECR1* and *GSTO1*) have been previously associated with IUGR (in animals and singleton humans, respectively), suggesting (at least in part) a common pathogenic mechanism for IUGR in singletons versus that in MC growth-discordant twins [17, 18].

In general, for each DMR, the patterns of DNAm differences in IUGR (Δβ) were consistent across the majority of twin pairs, although the magnitude of Δβ across different twin pairs varied (Additional file 1: Figure S9). Overall, this heterogeneity across the twin pairs is not unusual for either MZ twin studies [14] or for IUGR studies [19].

The IUGR twins in our series exhibited heterogeneity of IUGR placental pathology (Additional file 1: Table S2), which has been shown in other larger scale studies as well [12] and may relate to both cause and/or effect of the heterogeneity in the results of our epigenetic analysis.

### Prominent biological networks in IUGR

Our study demonstrates an association of IUGR with DNA methylation changes that impact the regulation of genes relating to lipid metabolism and to nervous system development. Interestingly, neonates with IUGR exhibit reduced lipid stores with significant neonatal metabolic morbidity [20] and are known to suffer neurodevelopmental difficulties compared to age-matched controls [21]. Our results implicate a role for altered DNA methylation in the cause and/or effect of these two major IUGR-related clinical impairments.

The largest methylation differences were found in the promoter region of the *DECR1* gene. This gene encodes DECR, the rate-limiting mitochondrial enzyme in unsaturated fatty acid beta oxidation, which is required for growth and neurodevelopment [22] and has also been implicated in adaptive cellular stress-response mechanisms in IUGR animals [17]. An association between unsaturated fatty acids and intrauterine growth has been previously reported in that prenatal supplementation of the unsaturated fatty acid, omega-3, has been shown to increase birth weight [23], while impaired beta oxidation in fetuses with IUGR has also been suggested by several other studies [17, 24, 25]. Biochemically diagnosed DECR deficiency has been reported in two unrelated individuals, who had poor growth, hypotonia, developmental delay, and severe encephalopathy; one of these individuals died in infancy [26, 27]. Studies of *Decr1*^−/−^ mice suggest that the enzyme is responsible for the induction of gluconeogenesis during fasting [28].

The next largest methylation difference identified in our analysis was a gain of methylation in the promoter of the *ZNF300* gene in the IUGR group compared to controls. *ZNF300* encodes a zinc finger protein, which functions as a ubiquitous transcriptional repressor [29]. Hypermethylation of this gene has been associated with decreased gene expression, modulation of inflammatory pathways, and tumor development [30]. *ZNF300* knockdown studies revealed this gene’s role in cell differentiation [31]. Although *ZNF300* has not previously been associated with IUGR, its strong role in cancer development supports its potential to impact fetal growth.

Another highly ranked candidate gene is the *LEPR* gene, which encodes the leptin receptor. LEPR deficiency in humans leads to morbid obesity and pituitary dysfunction [32–34], and polymorphisms in the *LEPR* gene have been associated with birth weight [35], obesity [36], and type II diabetes [37]. In animal studies, maternal diet restriction has been associated with subsequent obesity at a few months of age as well as reduced birth weight of the offspring and reduced expression of LEPR [38]. *Lepr*-deficient (Lepr db/db) mouse models are often used for studying type II diabetes (reviewed in [39]). To date, our study is the first to link increased *LEPR* methylation with IUGR in humans.

One previous study of growth-discordant MZ-MC twins assessed placental expression of 88 angiogenesis-related genes, the leptin (*LEP*) and *LEPR* genes, and also assessed methylation using an assay targeting 64 CpGs overlapping the *LEP* promoter [12]. They found that the smaller twins exhibited a significant increase in *LEP* expression along with an average gain of methylation of the *LEP* promoter region [12]. Four twin pairs used in that study were also used in our study (see the “Methods” section and Additional file 1: Tables S1–S2). It was difficult to compare Schrey et al.’s [12] data to our data as only six of the 63 CpG sites tested in the *LEP* promoter region are covered by the Illumina 450k array. Our analyses of the six CpGs for the eight MZ twin pairs and the four twin pairs overlapping the two studies did not show any significant gain of methylation in the *LEP* promoter region. That is, we suggest that the significant differences in *LEP* methylation found by Schrey and colleagues [12] were driven by CpGs not assessed in our study. The Schrey study also reported a decrease in *LEPR* expression in the IUGR group, though this difference was not significant [12]. A decrease in *LEPR* expression would be consistent with the gain of *LEPR* methylation found in the current study. In general, however, differences in the results of our study and those found by Schrey and colleagues [12] may largely be attributed to the use of different methodologies: genome-wide DNA methylation with limited coverage at individual promoter regions in this study, and gene expression analysis by Schrey and colleagues.

The remainder of the genes with methylation differences identified in the IUGR group (*DNAJA4*, *CCL28*, *HSPA1A/L*, *GSTO1*, and *GNE*) function in growth, immunity, and cell metabolism. The *GSTO1* gene, which encodes an arsenic detoxification enzyme, glutathione S-transferase omega 1, is the only gene in this list to have a prior association with fetal growth restriction [18]. This enzyme also plays a role in the inflammatory response, apoptosis, and tumor development (reviewed in [40]). The mouse *Gsto1* knockout model shows impaired detoxification of arsenic, a known human carcinogen [41]. *DNAJA4* and *HSPA1A/L* encode heat-shock proteins. A variety of tumors exhibit hypermethylation of *DNAJA4*, which has been associated with gene silencing [42, 43]. Increased expression of *DNAJA4* has been shown to suppress tumor invasion [42–44]. *CCL28*, which encodes the growth factor chemokine ligand 28 [45], increases placental cell apoptosis [46] and is downregulated in a variety of human malignancies [47, 48]. *GNE* encodes a key enzyme in sialic acid biosynthesis, a process that is often upregulated in tumor development [49]. Reduced sialylation has been shown in heterozygous *Gne*-deficient mice, while complete knockouts are embryonically lethal [50]. *Gne*-deficient mouse embryonic stem cells exhibit impaired proliferation and cell differentiation, particularly affecting muscle [51]. In humans, GNE deficiency causes Nonaka myopathy [52]. Collectively, the aberrant methylation of the genes found in our IUGR cohort appears to drive increased apoptosis, impaired cellular metabolism as well as poor growth, and invasion of the placenta.

### Prominent cellular pathways in IUGR

The Wnt and cadherin pathways were significantly over-represented in the list of candidate genes generated from 296 individual CpGs representative of the IUGR epigenome. Wnt signaling is well known for its role in carcinogenesis and embryonic development, specifically body axis patterning, cell proliferation, cell migration and cell differentiation [53, 54]. Wnt signaling plays a role in placental development (trophoblast proliferation and invasion) and is altered in gestational diseases (reviewed in [55]). Our group has previously shown that the promoter region of the *WNT2* gene is differentially methylated in the placentas of IUGR singletons versus controls [19]. *WNT2* gene expression has also been proposed to mediate poor maternal nutrition and fetal growth restriction [56]. Our results suggest that upregulation of *DNAJA4* may be an important downstream effect of impaired Wnt signaling in IUGR. Cadherins are a group of “calcium-dependent adhesion” transmembrane proteins that facilitate normal embryonic tissue organization and trophoblast invasion via cell-to-cell adhesion and cell migration (reviewed in [57]).

The precise mechanisms by which Wnt and cadherin signaling play a role in IUGR is unknown. To the best of our knowledge, this is the first report that prominently highlights these pathways in the pathogenesis of IUGR in growth-discordant MC twins.

## Conclusions

This is the first report of a genome-wide DNA methylation analysis using the Infinium HumanMethylation450 BeadChip platform to investigate IUGR in MC growth-discordant twins. We identified eight candidate genes that are epigenetically altered in IUGR. Our results suggest that dysregulation of lipid metabolism and transcription, as well as impaired cadherin and Wnt signaling, are associated with IUGR. Collectively, the genes exhibiting differential regulation in growth-restricted fetuses may underlie fetal programming and may generate candidate biomarkers for IUGR and/or its related adult-onset diseases. These results should be validated by further investigation of DNA methylation and/or gene expression of the identified candidate genes in a large cohort of MZ-MC growth-discordant twins. Finally, we propose the growth-discordant MC twin model as a useful tool in the genome-wide study of IUGR.

## Methods

### Sample selection

This study was approved by the Mount Sinai Hospital (MSH) and the Hospital for Sick Children (SickKids) Research Ethics Boards (REB # 10-0128-E). Participants were recruited from the MSH high-risk obstetrical clinic, following diagnosis of MC discordant growth and placental insufficiency. Informed consent was obtained from each participant. Growth discordance was defined as a birth weight difference >20 % between the co-twins [58]. Birth weight was plotted as percentile for gestational age using a Canadian population-based reference chart [59]. Placental insufficiency was defined as intermittent absent or reversed end-diastolic flow. A full list of the inclusion and exclusion criteria is found in Additional file 1: Table S6.

Following these criteria, we recruited eight MC growth-discordant twin pregnancies.

Placental characteristics are detailed in Additional file 1: Table S2. Four of these pairs overlap with a previous MZ twin study of IUGR that evaluated expression of angiogenesis-related genes and targeted DNA methylation of one gene [12].

Placental sampling was performed immediately after delivery by members of the Research Center for Women’s and Infants’ Health BioBank, Toronto. At delivery, cord clamps were labeled as twin “A” or “B” to distinguish each twin’s placental share. Snap-frozen placental samples were obtained from each twin according to a previously standardized protocol (http://biobank.lunenfeld.ca/). The remainder of each placenta was sent for pathological examination. Monozygosity was molecularly confirmed for all twin pairs.

### Genome-wide DNA methylation analysis

Genomic DNA from the placental samples was bisulfite treated (Qiagen). DNA methylation was then analyzed using the Infinium HumanMethylation450 BeadChip array (Illumina, San Diego, CA, USA) following the standard Infinium HD Assay Methylation Protocol Guide (Part #15019519, Illumina). The array assesses DNA methylation at single CpG sites genome-wide; however, it does not differentiate between 5-methylcytosine and 5-hydroxymethylcytosine, which is a recognized limitation of this platform [60].

All samples were run on the array together within the same batch and passed quality control. Data pre-processing such as background subtraction and normalization to internal array controls was performed in Illumina GenomeStudio v2011.1 Methylation Module 1.9 software (http://www.illumina.com).

### CpG probe filtering

The 485,577 methylation probes from the HumanMethylation450 array were filtered by removing probes with missing DNA methylation values (beta values either missing or exactly zero) and with detection *p* values above the significance threshold 0.05. We also removed all cross-reactive probes annotated as such in [61]. To minimize possible confounding effect by ethnicity or sex, especially in the context of unpaired statistical tests between IUGR and control groups (Fig. 6), all probes for which there was a known SNP of at least 1 % minor allele frequency within 5 bp of the targeted cytosine (based on the annotation in [61]) as well as probes located on sex chromosomes were excluded, leaving 399,118 autosomal CpG sites available for analysis.

### Detection of differentially methylated regions

To detect the differentially methylated regions, we used the bump hunting method of Jaffe and colleagues [15] implemented in the *bumphunter* Bioconductor package, setting the cutoff threshold of 10 % for the Δβ methylation difference in IUGR. To account for confounding effects, the model design matrix consisted of an intercept term, IUGR indicator variable, and twin sex, maternal age and twin’s gestational age as additional confounders. Statistical significance of the detected bumps was assessed in comparison to *B* = 1000 randomized bootstrap iterations. From the resulting collection of bumps, we retained only the DMRs that contained at least three CpGs with gaps no more than 500 bp, and  exhibited methylation differences of 10 % or higher in IUGR while satisfying the significance level *p* < 0.05.

### Selection of CpG sites for functional enrichment analysis

For the analysis of methylome-wide functional enrichment in IUGR, we included not only the eight DMRs from the bump hunting analysis, but also the CpG sites that were significantly different in IUGR compared to healthy co-twins. These individual CpG sites were identified using a combination of statistical criteria to account for the small sample size.

Three sets of statistical tests were performed on eight growth-discordant twin pairs at each CpG site. First, we used *limma* regression modeling, which is particularly well suited for small sample sizes thanks to its methodology of information borrowing across CpG sites. The model was applied to DNAm data log-transformed into M-values to improve statistical accuracy [62]. The twin’s sex, gestational age and maternal age were included in the model as covariates. CpGs were considered significant if the *limma* regression *p* values corresponding to the IUGR status satisfied the significance level *p* < 0.05.

Second, we used a non-parametric approach to test the between-group differences, comparing the placental methylation levels in the group of eight healthy individuals (controls) and the group of eight affected IUGR individuals. The unpaired Mann–Whitney *U* test was used for each CpG site.

Finally, the changes in DNA methylation level in healthy twins versus their IUGR-affected counterparts were compared using the paired Wilcoxon signed-rank test, which attempted to identify a consistent pattern of DNAm shift across all eight pairs, although not necessarily an overall difference between the IUGR and the healthy cohorts. Thus, the two non-parametric tests are complementary in finding pattern of differences across the twin pairs.

Given the large number of probes tested and a relatively small sample size, the standard methods of correction for multiple testing (such as Bonferroni or Benjamini-Hochberg FDR procedures) were too restrictive to confirm the validity of any of the individual tests. Therefore, we decided on a pragmatic strategy of using the probe-level statistical tests as guidance and complemented them with an effect-size threshold. We considered the threshold of 10 % as sufficiently stringent because relatively few CpG sites had a DNA methylation change exceeding 10 % by magnitude.

For validation purposes, we used unsupervised clustering to confirm that the resulting set of CpG sites contained a sufficiently discernible biological signal unobscured by the potential false positives or other stochastic noise in the selected loci. All DNAm profiles were first reduced to the set of probes with significant differences in IUGR according to our selection criteria. Thereafter, hierarchical clustering was applied to discern the different groups in our cohort.

### Functional enrichment analysis

To identify prominent clinical mechanisms affecting and/or affected by growth restriction in our cohort, the 172 genes with evidence of differential methylation were investigated with respect to their functions and major biological roles. The analysis was performed using DAVID [63]. All *p* values were FDR corrected.

The 296 putatively affected CpG set was also analyzed in the context of wider genomic regions using GREAT [64]. We set the distal extension to the nearest genes as 100 kbp. The background set of probes to which the comparison was made was defined as the 399,118 autosomal CpGs used as the initial input to our analysis pipeline.

### Validation analysis of DNA methylation using bisulfite pyrosequencing

Results of top candidate CpG sites (cg01971612 and cg18485485 for *DECR1*; cg04675542, cg02343823, and cg08580836 for *ZNF300*; and cg08234308 for *LEPR*) were validated using pyrosequencing of bisulfite converted DNA (PyroMark Q24; Qiagen). Polymerase chain reaction (PCR) and sequencing primers were designed using PyroMark Assay Design 2.0. All procedures were performed according to the manufacturer’s protocols [65]. The presence of 5-methylcytosine versus 5-hydroxymethylcytosine could not be distinguished using this method.

## Abbreviations

DMR, differentially methylated region; DNAm, DNA methylation; FDR, false discovery rate; IUGR, intrauterine growth restriction; MC, monochorionic; MZ, monozygotic; PCR, polymerase chain reaction

## Additional files

Additional file 1: Supplementary Figures and Tables. (PDF 1827 kb) Additional file 2: Supplementary **Tables S3**–**S5**. (XLSX 86 kb)
