# Supplementary material for: Genome-wide placental DNA methylation analysis of severely growth-discordant monochorionic twins reveals novel epigenetic targets for intrauterine growth restriction
Source: Clin Epigenetics. 2016 Jun 21;8:70. doi: 10.1186/s13148-016-0238-x (PMC4915063; doi:10.1186/s13148-016-0238-x)
Supplement: Additional file 1: — Supplementary Figures and Tables. (PDF 1827 kb) [file 13148_2016_238_MOESM1_ESM.pdf]

Genome-wide placental DNA methylation analysis of severely growth-discordant monochorionic twins reveals novel epigenetic targets for intrauterine growth restriction

Supplementary Materials

SUPPLEMENTARY FIGURES

Figure S1. **Differentially methylated regions associated with IUGR.** The DMR is associated with the large difference between IUGR and Control, which can be either loss (green boxes, panels **A-B**) or gain (red boxes, panels **C-E**) of DNA methylation. Other genomic features, such as CpG islands, histone marks, DNase I hypersensitivity clusters and transcription factor binding sites are also shown. Visualization is provided by the UCSC Genome Browser.

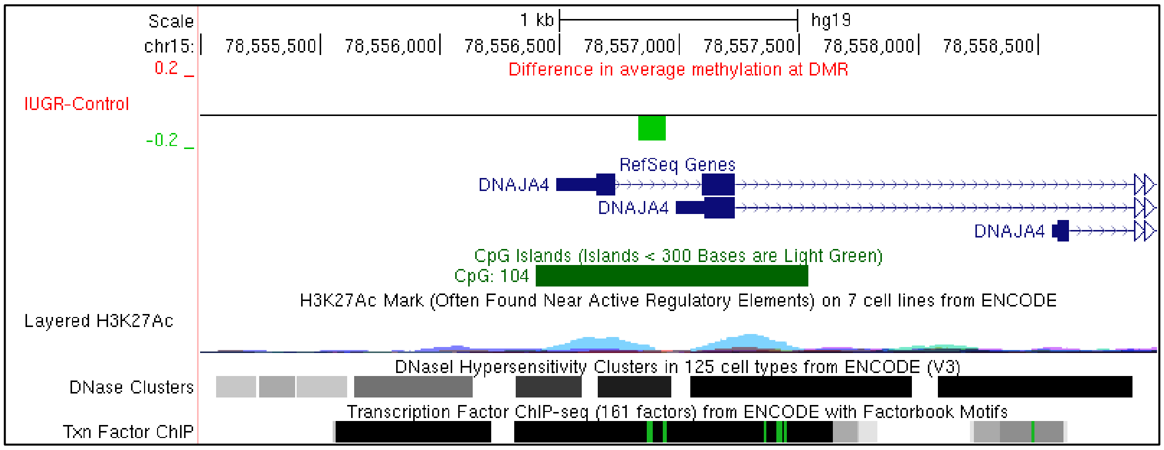

A

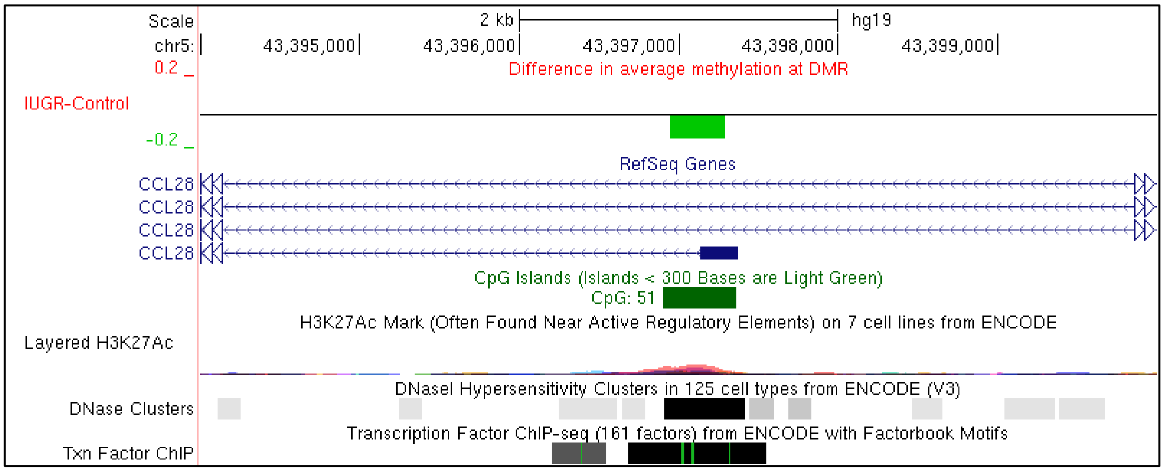

B

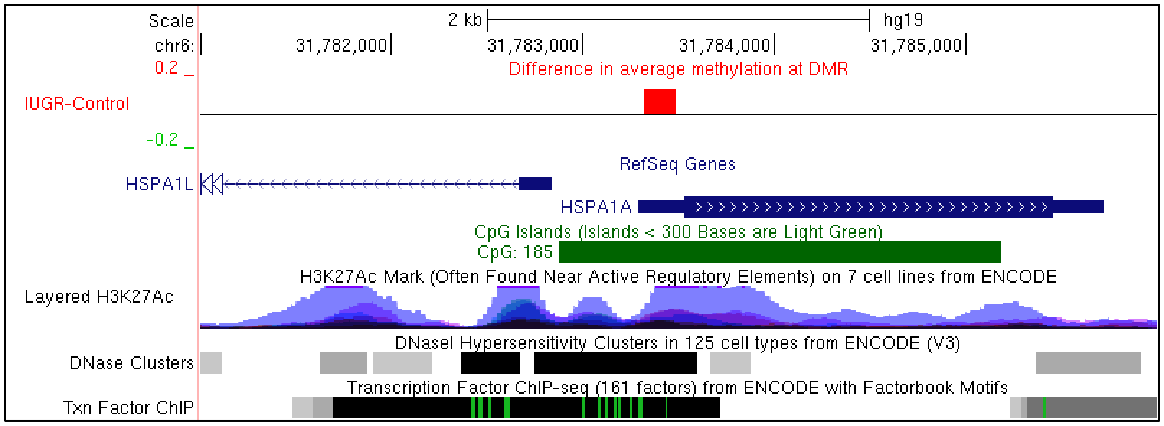

Scale
2 kb
hg19
chr6:
31,782,000
31,783,000
31,784,000
31,785,000
0.2 _
Difference in average methylation at DMR
IUGR-Control
-0.2 _
RefSeq Genes
HSPA1L
HSPA1A
CpG Islands (Islands < 300 Bases are Light Green)
CpG: 185
H3K27Ac Mark (Often Found Near Active Regulatory Elements) on 7 cell lines from ENCODE
Layered H3K27Ac
DNaseI Hypersensitivity Clusters in 125 cell types from ENCODE (V3)
DNase Clusters
Transcription Factor ChIP-seq (161 factors) from ENCODE with Factorbook Motifs
Txn Factor ChIP

C

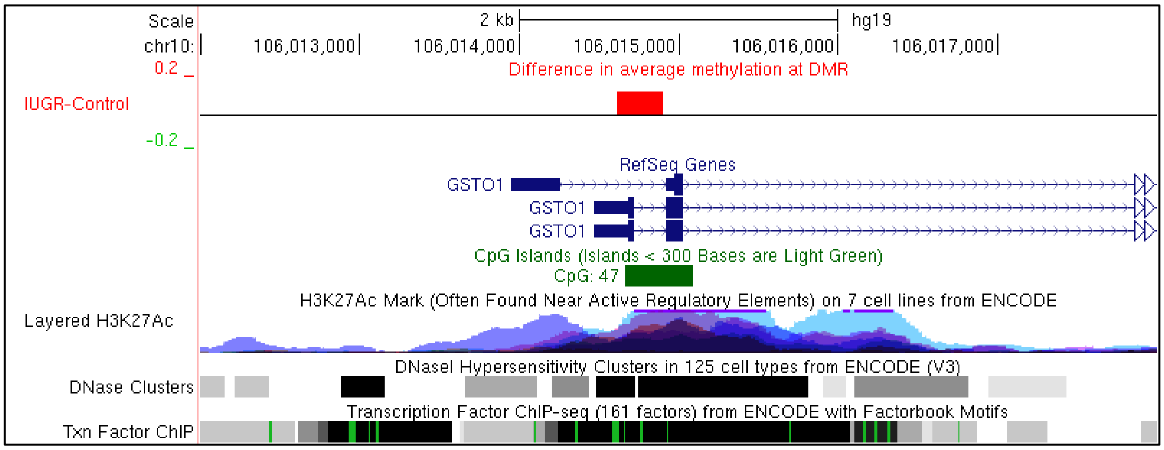

Scale
2 kb
hg19
chr10:
106,013,000
106,014,000
106,015,000
106,016,000
106,017,000
0.2 _
Difference in average methylation at DMR
IUGR-Control
-0.2 _
RefSeq Genes
GSTO1
GSTO1
GSTO1
CpG Islands (Islands < 300 Bases are Light Green)
CpG: 47
H3K27Ac Mark (Often Found Near Active Regulatory Elements) on 7 cell lines from ENCODE
Layered H3K27Ac
DNaseI Hypersensitivity Clusters in 125 cell types from ENCODE (V3)
DNase Clusters
Transcription Factor ChIP-seq (161 factors) from ENCODE with Factorbook Motifs
Txn Factor ChIP

D

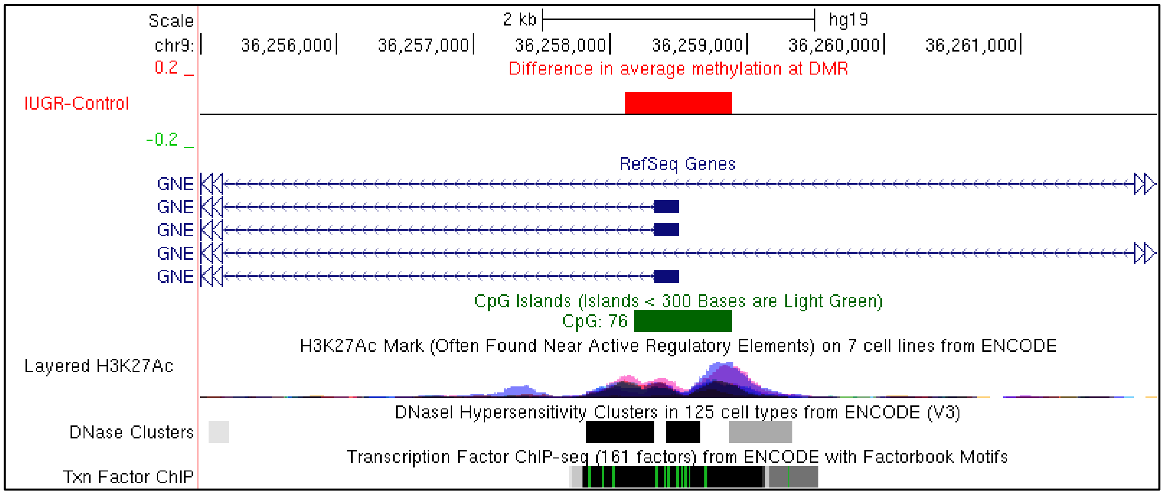

Scale
2 kb
hg19
chr9:
36,256,000
36,257,000
36,258,000
36,259,000
36,260,000
36,261,000
0.2 _
Difference in average methylation at DMR
IUGR-Control
-0.2 _
RefSeq Genes
GNE
GNE
GNE
GNE
GNE
CpG Islands (Islands < 300 Bases are Light Green)
CpG: 76
H3K27Ac Mark (Often Found Near Active Regulatory Elements) on 7 cell lines from ENCODE
Layered H3K27Ac
DNaseI Hypersensitivity Clusters in 125 cell types from ENCODE (V3)
DNase Clusters
Transcription Factor ChIP-seq (161 factors) from ENCODE with Factorbook Motifs
Txn Factor ChIP

E

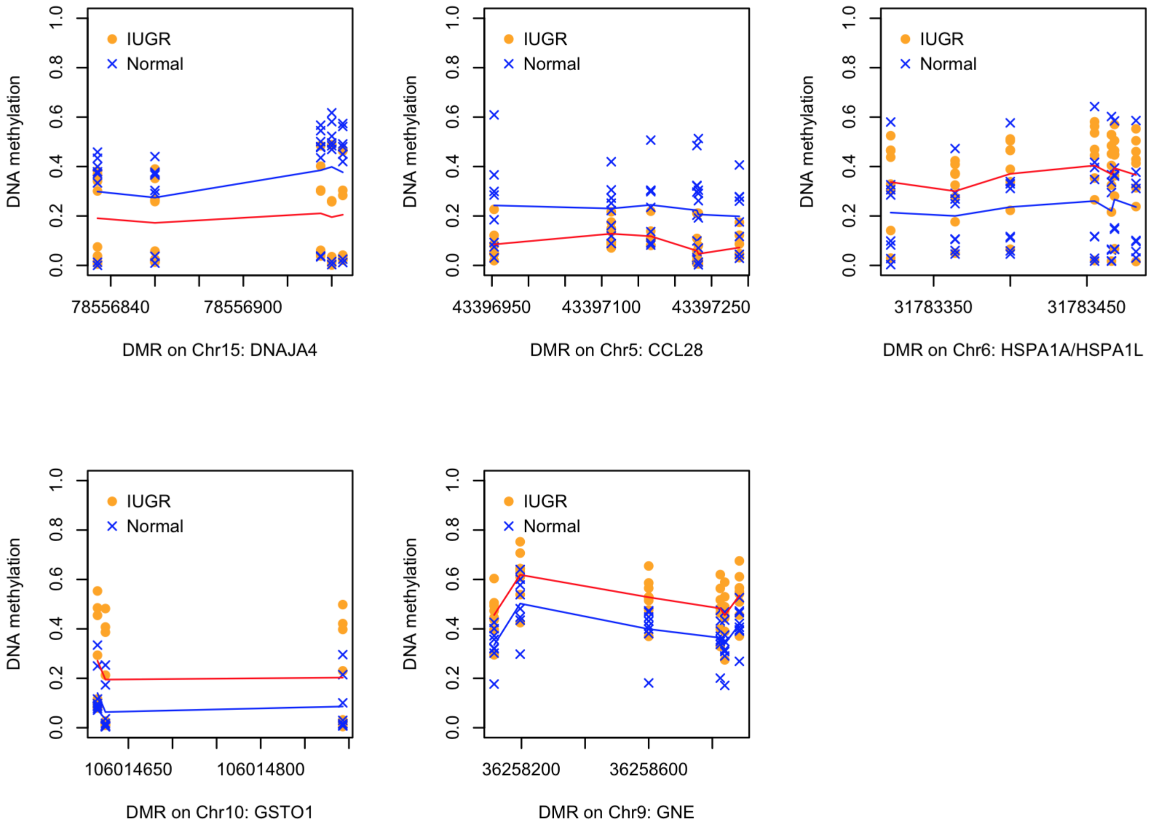

Figure S2. DNAm changes in IUGR twins in CpGs comprising the detected differentially methylated regions (DMRs). Methylation levels in IUGR twins (orange circles) and unaffected co-twins (blue crosses) are shown, arranged vertically for each CpG in the region spanning the candidate gene promoter. The mean methylation levels are shown for the IUGR cohort (red line) and controls (blue line). All CpGs exhibit methylation change above 10%, corresponding to the wider gap between the red and blue lines. Visualization is inspired by DMRcate software (Peters et al., 2015).

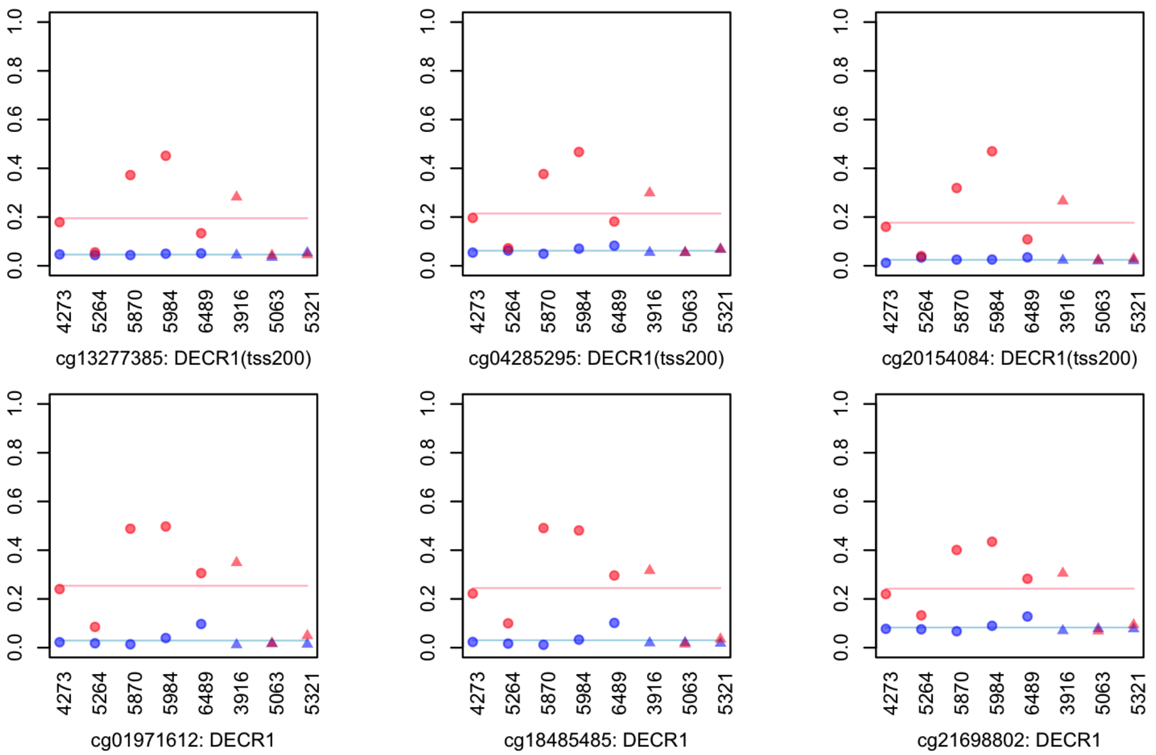

Figure S3. CpGs that comprise the DMR in *DECR1* promoter region. The X-axis shows 8 twin pairs, with the methylation level on the Y-axis for the healthy (blue) and the affected (red) twins. Horizontal lines show the average DNAm levels for the 8 healthy twins (blue lines) and 8 affected twins (red lines). The sex is indicated using shapes (male = circles, female = triangles). 'tss200' and 'tss1500' indicate the regions 200 bp or 1500 bp upstream of the transcription start site (according to Illumina annotation).

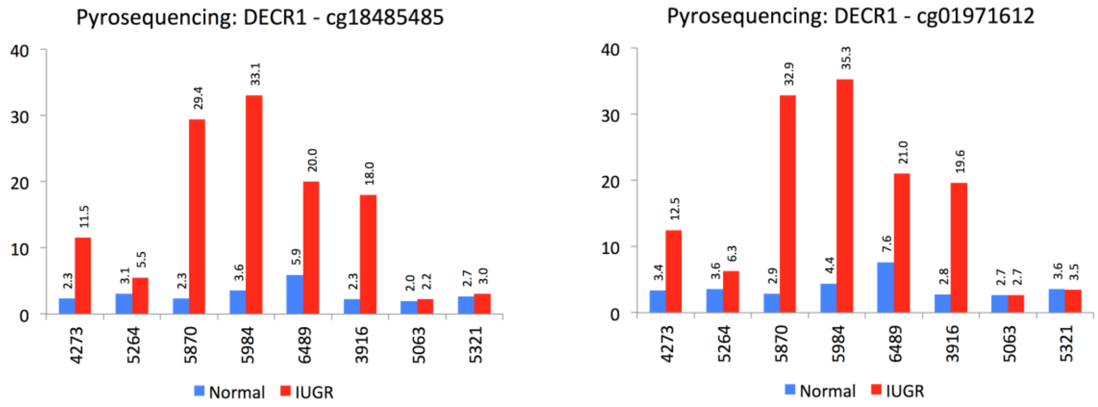

Figure S4. Validation of *DECR1* methylation by pyrosequencing. The pyrosequencing results for the top two DECR1 CpG sites from the IUGR signature confirm the methylation patterns detected using the Infinium HumanMethylation450 array (compare to the **Supplementary Figure S3** above). X-axis shows twin samples, Y-axis shows the methylation percentage (beta values).

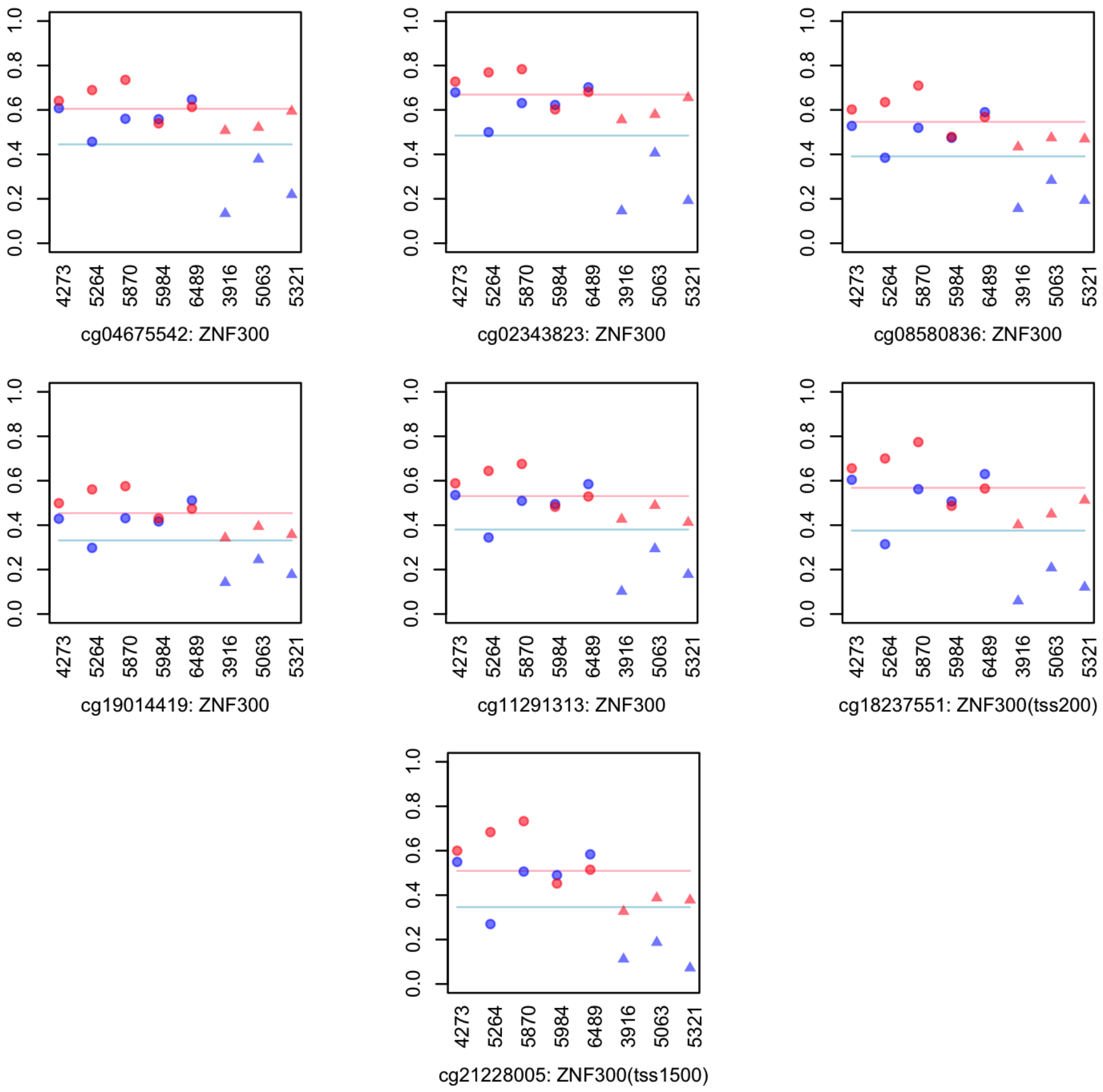

Figure S5. CpGs that comprise the DMR in *ZNF300* promoter region. The X-axis shows 8 twin pairs, with the methylation level on the Y-axis for the healthy (blue) and the affected (red) twins. Horizontal lines show the average DNAm levels for the 8 healthy twins (blue lines) and 8 affected twins (red lines). The sex is indicated using shapes (male = circles, female = triangles). 'tss200' and 'tss1500' indicate the regions 200 bp or 1500 bp upstream of the transcription start site (according to Illumina annotation).

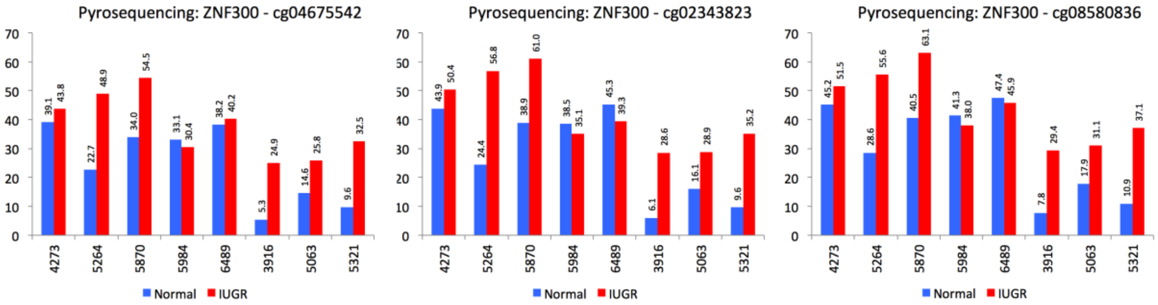

Figure S6. Validation of *ZNF300* methylation by pyrosequencing. The pyrosequencing results for three *ZNF300* CpG sites from the IUGR signature confirm the methylation patterns detected using the Infinium HumanMethylation450 array (compare to the **Supplementary Figure S5** above). X-axis shows twin samples, Y-axis shows the methylation percentage (beta values).

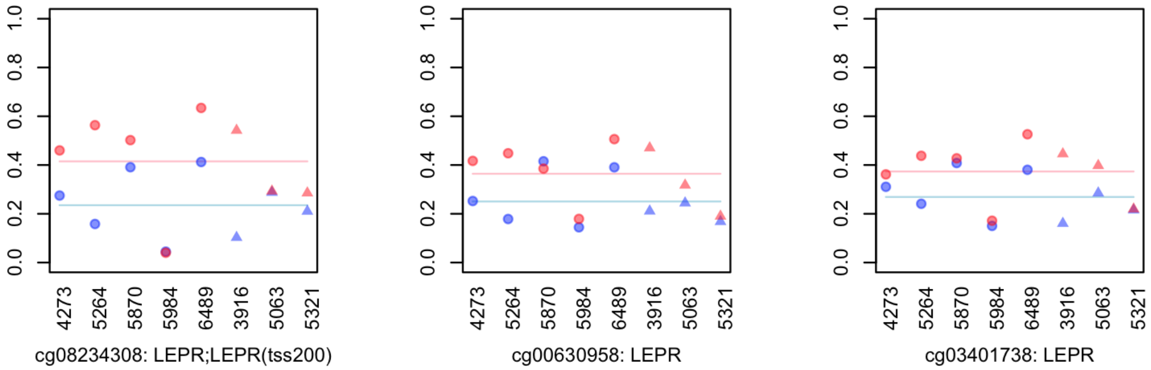

Figure S7. CpGs that comprise the DMR in *LEPR* promoter region. The X-axis shows 8 twin pairs, with the methylation level on the Y-axis for the healthy (blue) and the affected (red) twins. Horizontal lines show the average DNAm levels for the 8 healthy twins (blue lines) and 8 affected twins (red lines). The sex is indicated using shapes (male = circles, female = triangles). 'tss200' and 'tss1500' indicate the regions 200 bp or 1500 bp upstream of the transcription start site (according to Illumina annotation).

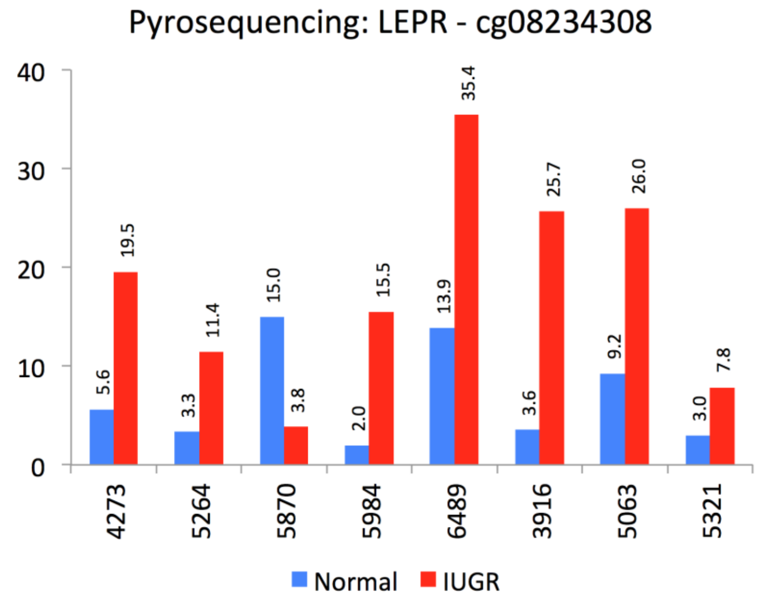

Figure S8. Validation of *LEPR* methylation by pyrosequencing. The pyrosequencing results for the top *LEPR* CpG site from the IUGR signature confirm the methylation patterns detected using the Infinium HumanMethylation450 array (compare to the **Supplementary Figure S7** above). X-axis shows twin samples, Y-axis shows the methylation percentage (beta values).

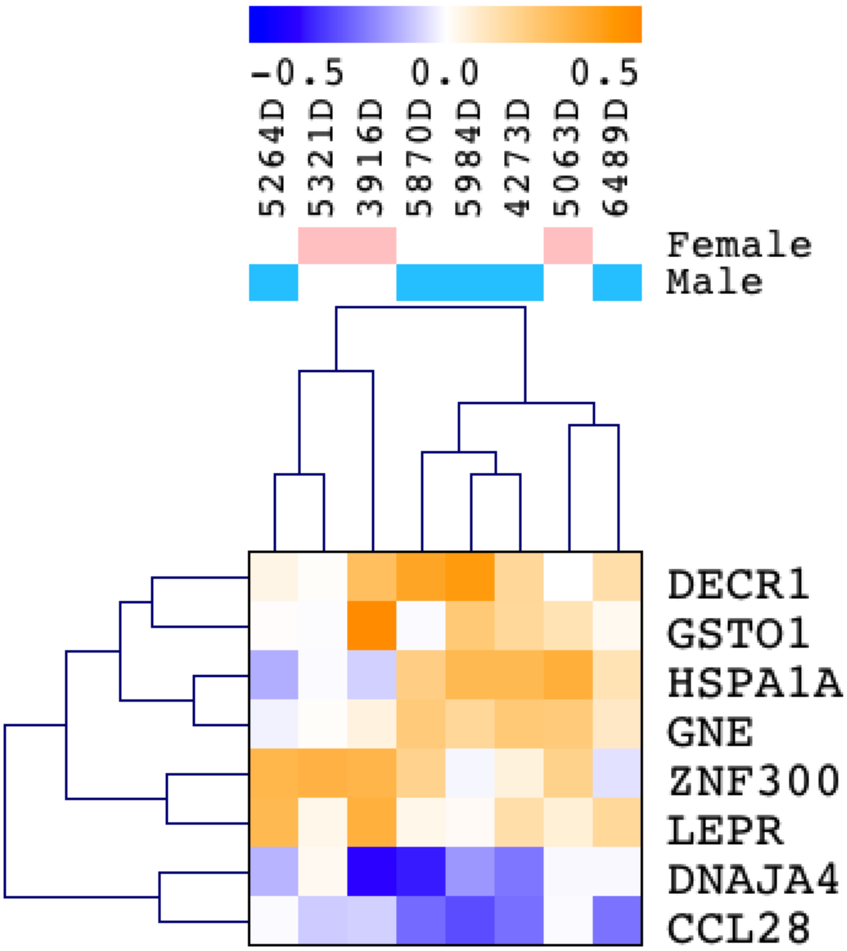

Figure S9. **Variation of DNA methylation changes in differentially methylated regions (DMR) across different twin pairs.** For each of the eight DMRs (heatmap rows) and 8 twin pairs (columns), the heatmap shows the average methylation change in IUGR across all CpGs that comprise the DMR. Each value of the heatmap represents a DNAm gain (orange) or loss (blue) in the IUGR twin of the pair, according to the color scale shown at the top.

SUPPLEMENTARY REFERENCES

Peters, T. J., et al. (2015). ‘De novo identification of differentially methylated regions in the human genome.’ *Epigenetics & Chromatin*, 8, 6.

Redline, R. W. (2008), 'Placental pathology: a systematic approach with clinical correlations', *Placenta,* 29 Suppl A, S86-91.

Schrey, S., et al. (2013), 'Leptin is differentially expressed and epigenetically regulated across monochorionic twin placenta with discordant fetal growth', *Mol Hum Reprod,* 19 (11), 764-72.

SUPPLEMENTAL TABLES

Twin pair ID no.	Twin pair sex	Maternal age	Ethnicity	Mode of conception	Larger twin birth weight (g)	Smaller twin birth weight (g)	Birth weight discordance (%)	Gestational age (weeks+day)
3916D	F	33	Chinese	Spont	2350	1400	40.4	34+6
4273D*	M	29	Indian sub-continent	Spont	1280	680	46.9	28+5
5063D*	F	31	Indian sub-continent	Spont	1910	1440	24.6	33
5264D*	M	41	Guyanese	Spont	2640	1720	34.8	34+3
5321D*	F	28	Caucasian	Spont	2480	1550	37.5	34+1
5870D	M	31	Caucasian	Spont	2370	1360	42.6	33+3
5984D	M	36	Caucasian	IVF	1000	790	21	28+1
6489D	M	31	Caucasian	Spont	1280	520	59.4	30+2

Table S1. Twin sample characteristics. Spont = spontaneous conception; IVF = in vitro fertilization. Birth weight discordance calculated as: (larger twin weight – small twin weight)/larger twin weight. * = twin pair from (Schrey et al. 2013)

Twin pair ID no.	Placental weight (g)	Placental weight percentile	Placental share of smaller twin (%)	Smaller twin's placental pathology	Smaller twin's cord insertion
3916D	637.5	10-25th	33	Advanced villous maturity	Vel
4273D*	430	25-50th	33	Fetal vascular thrombosis, avascular villi, chronic villitis and deciduitis	Vel
5063D*	361	<10th	50	Advanced villous maturity	N
5264D*	702.2	25-50th	25	None	N
5321D*	721.9	25-50th	40	None	Vel
5870D	680	50th	15	Focal infarction; cord overcoiling	Vel
5984D	274	<10th	50	None	N
6489D	641	75-98th	25	Infarction	Vel

Table S2. Placental characteristics of discordant MZ-MC twin pairs. One-centimeter sections of placental parenchyma, cord and membrane from each twin were examined for pathological features and abnormal morphology (Redline 2008). Placental weights were plotted against norms for gestational age (Redline 2008). Cord insertion: N = normal, Vel = velamentous, * = twin pair from (Schrey et al. 2013).

**Inclusion Criteria**
Difference in birth weight ≥ 20% for weight of larger twin
Informed consent of patient
**Exclusion Criteria**
Twins with evidence of twin-to-twin transfusion syndrome (TTTS) were excluded from this group (defined as either poly/oligohydramnios-sequence or absence of bladder in the small twin and enlarged bladder in the larger twin
Twin anemia-polycythemia sequence (TAPS)
Monochorionicity not confirmed on pathology
Chromosomal abnormalities/ congenital syndromes
Fetal abnormalities
Intrauterine fetal demise (IUFD) / neo-natal death (NND)
Congenital infection
Preeclampsia
Gestational or pre-existing diabetes
Placental tumors, such as chorangioma on pathology exam
Fetal alcohol syndrome or drug abuse
Maternal disease not controlled for in both groups (asthma, flu, depression, celiac disease)

Table S6. Inclusion and exclusion criteria for the twin samples.
